# A Cross-Sectional Study of Human Immunodeficiency Virus-Associated Neurocognitive Deficit in Central India

**DOI:** 10.7759/cureus.18776

**Published:** 2021-10-14

**Authors:** Dr. Saptarshi Maitra, Mrinalini Motlag

**Affiliations:** 1 Internal Medicine, Indira Gandhi Government Medical College, Nagpur, IND

**Keywords:** hiv neuroinfections, dementia, people living with hiv/aids, hiv aids, hiv-associated neurocognitive disorder

## Abstract

Background

With the advent of modern era of combination antiretroviral therapy (cART) and increased longevity of people living with human immunodeficiency virus (PLHIV), human immunodeficiency virus-associated neurocognitive disorder (HAND) is commonly observed. This study explores the prevalence of HAND and the demographic and treatment variables in people with HAND, in Central India.

Research methodology

PLHIV on cART visiting HIV clinic underwent screening for substance abuse using CAGE-AID, and depression using PHQ-2 followed by PHQ-9. The screening rules out overt conditions which might interfere with cognitive abilities of the individual and thereby act as confounding factor. Thus, a sample population of 96 was obtained, on whom International HIV Dementia Scale (IHDS) was applied to screen for dementia. Out of 96, 16 individuals detected to suffer from HAND. Quality of Life was assessed by Patient’s Assessment of Own Functioning Inventory (PAOFI).

Results

Prevalence of HAND was estimated to be 16/96 (16.66%). It was more common amongst unmarried individuals (p < 0.001) and lower educational status (p < 0.01) among social variables; while shorter duration of ART (<3 years) (p < 0.01) and lower CD4 nadir (≤200 cell/mm^3^; p<0.01) showed significant correlation among clinical variables. PAOFI revealed significant association between HAND and quality of life (p-value < 0.01, CI = 95%). Modified Mental State Examination (3MS), which determines cognitive ability in various domains based on tasks, was mostly affected for - similarities and read and obey (for 43.75% population).

Conclusion

Social and clinical variables play a significant role in development of HAND. Routine screening for HAND in PLHIV will help in early identification and management of the disease. The quality of life for those suffering from the burden of HIV and HAND can be significantly improved if approached and treated early in the course of the disease.

## Introduction

The pandemic of the human immunodeficiency virus (HIV), first recognised in 1981, has engulfed the entire planet in less than four decades. The number of infected individuals was as high as 37.9 million, by the end of 2018, according to The Joint United Nations Programme on HIV and AIDS (UNAIDS) [1]. The majority (95%) of these people living with HIV (PLHIV) reside in low- and middle-income categories [1]. With the advent of the era of modern medicine and combination antiretroviral therapy (cART), we see a constant decline in morbidity and mortality. With increasing chronicity, novel manifestations of the disease are starting to unravel themselves. One of the major complications of HIV observed in chronic cases is HIV-associated neurocognitive disorder (HAND). HAND includes a spectrum of neurocognitive changes, involving personality, motor, and cognitive changes. It ranges from asymptomatic neurocognitive impairment (ANI) - where the patient has no deficits on neurocognitive tests or with activities of daily living (ADL) or instrumental activity of daily living (IADL), to HIV-associated dementia (HAD) which is a severe form of HAND, causing significant difficulties in performing tasks of daily living [2]. The cognitive decline caused by HIV, however mild, when coupled with age and other co-morbidities causes increased difficulty to independent living. Therefore, the early screening and management of this disorder provide an opportunity to prevent such a fate. This study aims at shedding light on the following topics: (i) to estimate the frequency of HAND in PLHIV on cART without co-morbidities; (ii) to compare social variables (gender, marital status, presence of caretakers) in individuals with HAND; (iii) to compare clinical variables (duration of cART and CD4 count nadir) in individuals with HAND; (iv) to identify the pattern of involvement of cognitive domains in PLHIV with and without HAND using the Modified Mini-Mental State Examination (3MS); (v) to assess the effect on daily activities in PLHIV with HAND using Patient’s Assessment of Own Functioning Inventory (PAOFI).

## Materials and methods

This cross-sectional study was carried out in Central India after the approval of the Institutional and Ethics Committee of Indira Gandhi Government Medical College and Mayo Hospital, Nagpur (IRB Approval Number - IGGMC/Pharmacology/IEC/68/2016). The inclusion criteria used for the sample population were - previously diagnosed PLHIV on cART for at least one year, age more than 18 years, International HIV Dementia Scale (IHDS) < 10, and willingness to provide informed consent.

While the exclusion criteria for the sample population were - current alcohol/drug abuse, systemic hypertension, diabetes mellitus, stroke, chronic kidney disease (CKD), chronic liver disease (CLD), and any psychiatric illness.

Participants were screened in accordance with the exclusion criteria and risk factors identified by Saylor et al. [3]. CAGE Adapted to Include Drugs (CAGE-AID) Questionnaire was used to eliminate those with alcohol/drug abuse [4,5]. Patient Health Questionnaire-2 (PHQ-2) was used for undiagnosed depression. Those who screened positive were further evaluated by PHQ-9. This tool allows for the screening, diagnosing, monitoring, and measuring of the severity of depression.

Ninety-six HIV cases were found fit to participate in the research study. The case record forms were duly filled. Individuals were classified on the basis of them having been educated up to the 12th standard or not [6]. IHDS, a four-item set of tests was completed by a clinician, include memory task, finger tapping, a sequential motor task (Luria Sequence), and recall [7]. The choice of using IHDS over Mini-Mental State Examination (MMSE) is substantiated by several studies including the one done by Oshinaike et al. [8].

Modified Mini-Mental State Examination was used to identify cognitive impairment in various domains. The cognitive domain examined were orientation, registration, mental reversal, first recall, temporal orientation, spatial orientation, naming, four-legged animals, similarities, repetition, read and obey, writing, copying two Pentagons, three-staged command,s and second recall. 

The PAOFI was administered to identify the impairment in the daily activities of the participants. Frascati criteria require >2SD affection in at least two cognitive domains in 3MS to qualify as HAND [2].

## Results

Distribution of demographic variables in the study population

The population was stratified on the basis of age and gender to understand the diversity. Table 1 shows the distribution of different participants based on age and gender. The highest number of participants belong to the age group of 41-50 years followed by 51-60 years.

**Table 1:** Age gender distribution of PLHIV on cART (n = 96) cART: combined antiretroviral therapy, PLHIV: people living with human immunodeficiency virus.

Age (years)	Males	Females	Total
20–30	8	2	10
31–40	10	5	15
41–50	17	19	36
51–60	16	11	27
>61	3	5	8

Stratifying the population on the basis of marital status, educational status, caretaker, and clinical variables (duration of cART and CD4 count) allows us to study the groups with their specific variable. As we can see in Table 2, the population studied has more married individuals (males - 42, females - 31) and is educated less than 12th standard (males - 35, females - 26). The discrepancies in the other variables are significantly less apparent.

**Table 2:** Distribution of PLHIV on cART according to social variables Std: standard, cART: combined anti-retroviral therapy, HAND: human immunodeficiency virus-associated neurocognitive deficit, CD 4: cluster of differentiation 4

Marital status	Married		Unmarried	
	Males	Females	Males	Females
	42	31	12	11
Educational status	Educated > 12^th ^std		Educated ≤12^th ^std	
	Males	Females	Males	Females
	19	16	35	26
Caretaker	Present		Absent	
	Males	Females	Males	Females
	26	27	28	15
Duration of cART	<3 years		≥3 years	
	Males	Females	Males	Females
	30	27	24	15
CD 4 count	<200 cells/mm^3^		≥200 cells/mm^3^	
	Males	Females	Males	Females
	15	16	39	26

Upon applying the Frascati principle, groups of individuals with and without HAND were formed. Most individuals being in the age group of 41-50 years, and having more male participants as shown in Table 3.

**Table 3:** Distribution of cases of HAND in the study population HAND: human immunodeficiency virus-associated neurocognitive deficit

Age (years)	Males	Females	Total
20–30	0	0	0
31–40	2	1	3
41–50	4	3	7
51–60	3	2	5
>61	0	1	1
Total			16

Comparison of social variables in relation to HAND

From Table 4, we can infer that HAND is more common among unmarried participants and those who had completed education beyond high school. Using the Chi-squared table, marital status (p < 0.001) and education status (p < 0.003) turn out to be of significance. The frequency of HAND was considerably less in married participants as compared to unmarried counterparts. The discrepancy is significantly lesser when it comes to the presence of caretakers in the study population.

**Table 4:** Distribution of individuals with HAND amongst various social groups Std: standard, HAND: human immunodeficiency virus-associated neurocognitive deficit

Marital status	Married		Unmarried	
	Males	Females	Males	Females
	1	2	9	4
Educational status	Educated >12^th ^std		Educated ≤12^th ^std	
	Males	Females	Males	Females
	3	1	7	5
Caretaker	Present		Absent	
	Males	Females	Males	Females
	5	4	5	2

Comparison of clinical variables in relation to HAND

Tables 5-6 illustrate the mean and the statistical significance of clinical variables. The tables infer a negative correlation between the presence of HAND and the duration of cART as well as CD4 nadir.

**Table 5:** Mean of clinical variables in study groups with and without HAND cART: combined anti-retroviral therapy, HAND: human immunodeficiency virus-associated neurocognitive deficit, PLHIV: people living with human immunodeficiency virus, CD4: cluster of differentiation 4

Mean duration of cART in PLHIV with HAND (in years)	Mean duration of cART without HAND (in years)
2.06 ± 0.87	2.48 ± 0.96
Mean CD4 nadir in PLHIV with HAND (cells/mm^3^)	Mean CD4 nadir in PLHIV without HAND (cells/mm^3^)
213.85 ± 57.15	273.40 ± 64.47

**Table 6:** Relationship of clinical variables (CD4 count nadir and duration of cART) with HAND cART: combined anti-retroviral therapy, CD4: cluster of differentiation 4, HAND: human immunodeficiency virus-associated neurocognitive deficit

Category	Chi-square value	dF value	P-value
CD 4 count Nadir	7.39	1	0.006
Duration of cART	7.08	1	0.007

Cognitive domain involvement in PLHIV with and without HAND using 3MS

**Figure 1:**
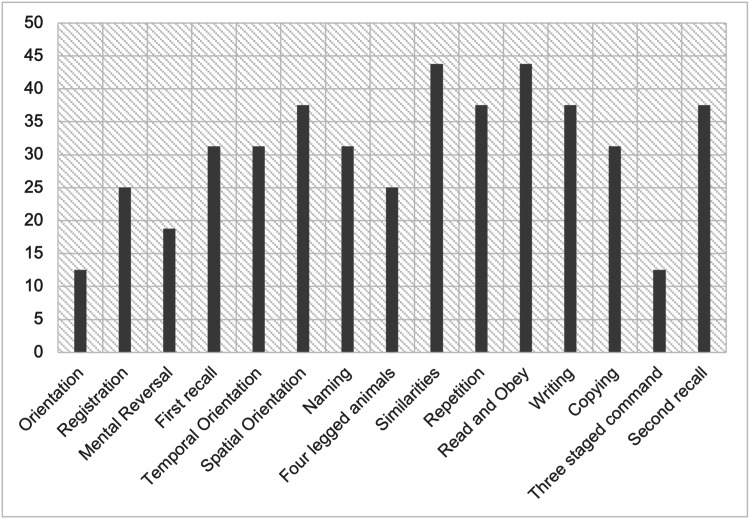
Involvement of cognitive domains in individuals with HIV HAND: human immunodeficiency virus-associated neurocognitive deficit, PLHIV: people living with human immunodeficiency virus

**Figure 2:**
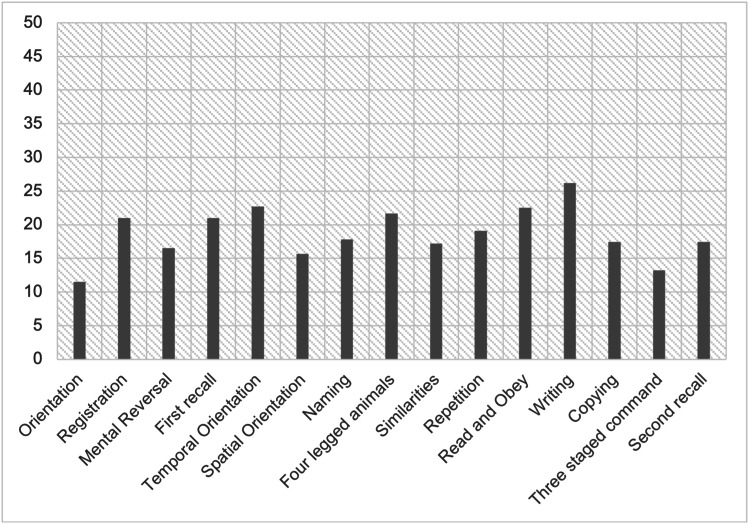
Involvement of cognitive domains in individuals without HIV HAND: human immunodeficiency virus-associated neurocognitive deficit, PLHIV: people living with human immunodeficiency virus

Amongst the various domains assessed under the 3MS, we can see a greater number of participants with HAND performing poorly than those without. Out of the 15 domains tested in PLHIV with HAND (Figure 1), similarities and read and obey were maximally affected at 43.75%. This is followed by spatial orientation, writing, repetition, and second recall at 37.5%. Orientation and three-staged command were least affected at 12.5%. In PLHIV without HAND (Figure 2), writing was most affected at 26.2%, while orientation was least affected at 11.5%.

Assessment of effect on daily activities in PLHIV with and without HAND using PAOFI

Table 7 below shows the difference in scores attained by participants with and without HAND on PAOFI. The overall Chi-square value of 12.09, p-value <0.00 for CI = 95%. The individual tests were found to have Chi-square values of 55.54 (Memory), 24.03 (Language and Communication), 45.22 (Motor), 75.23 (Sensory), and 39.30 (Higher function) arriving at a p-value of <0.01 with CI = 95%. Hence, the prediction of the test is significant for the deficit in all domains, and HAND can be strongly suspected in a positive test.

**Table 7:** Comparison of mean of scores of PAOFI in individuals with and without HAND HAND: human immunodeficiency virus-associated neurocognitive deficit, PLHIV: people living with human immunodeficiency virus, PAOFI: Patient’s Assessment of Own Functioning Inventory

Domains tested (total score)	Mean score in PLHIV with HAND	Mean score in PLHIV without HAND
Memory (60)	23.58 ± 12.36	58.13 ± 4.09
Language and communication (54)	21.29 ± 9.58	52.15 ± 4.15
Motor (12)	4.64 ± 2.89	11.67 ± 0.72
Sensory (18)	5.47 ± 3.44	16.54 ± 2.53
Higher functions (54)	20.70 ± 11.10	51.87 ± 8.26
The total score of PAOFI (168)	75.70 ± 19.65	190.37 ± 9.89

## Discussion

A cross-sectional study of 96 pre-diagnosed PLHIV on cART, satisfying the inclusion and exclusion criteria, were evaluated for HAND to study the association of social and clinical variables with the disease. The association of the social variables (Tables 1-4) namely age, marital status, educational status, caretaker, and clinical variables (Tables 5-6) namely duration of cART and CD4 count with HAND were studied. The cognitive domains involved in PLHIV and activities of daily living were assessed.

On applying the Frascati criteria to the study population, 16 participants (16.66%) were found to have HIV-associated neurocognitive deficits. The percentage of the affected population was similar to the CHARTER study at 22.7%; the study was done by Schouten et al. at 15-50% and Gisslén et al. at 15.9% [9-11].

From Table 1, we see that HAND was slightly higher amongst male participants (10), whereas it was noted in six female participants. This was not statistically significant. Kumar et al. have postulated that the disease load is high amongst males and they have proportionately greater utilisation of the health system [12].

From Tables 2-4, we observe that marital status and educational status have a significant negative association with HAND. Gender and the presence of caretakers held no such significant association. Support provided by spouse and family may have contributed to better cognition as married individuals performed better. Lower educational level is associated with neurocognitive impairment in a study done by Yusuf et al. in the Nigerian population [13]. We have hypothesised that the cause of the association of neurocognitive deficit with lower education could be poorer synaptic development. These synapses may then be more prone to damage by the viral particle.

In Tables 5-6, the duration of ART has been categorised between <3 and ≥3 years [9]. HAND was present in 12 participants who received ART for <3 years and in 4 who received it for ≥3 years. Lower CD4 nadir (<200 cell/mm^3^) shows a higher number of cases at 11 while ≥200 CD4 cell/mm^3^ is seen in five cases. The specific cut-off at 200 CD4 cell/mm^3^ nadir was taken in accordance with the CHARTER study findings [9]. A significant negative correlation was noted between the duration of ART and CD4 nadir with HAND. Studies performed by Kumar et al. and Njamnshi et al. identify a negative correlation between the duration of ART and CD4 nadir [12,14]. This negative association points towards the pathophysiology of the disease which suspects the transmission of viral protein across the blood-brain barrier causing damage to cortical tissue. The low CD4 nadir is used as an indirect marker for higher disease load. The higher disease load could be attributed to shorter duration cART or to the poorer compliance to medication due to absence of understanding owing to poor educational status.

On administration of Modified Mini State Examination to identify the pattern of affection of cognitive domains among HAND patients in the sample population, it is observed that there was a maximum reduction in similarities and read and obey at 43.75%. A study performed by Kumar et al. showed the maximum reduction in similarities at 48.3% of their sample population, while read and obey showed a reduction in only 24% of the population [12]. The minimum reduction, in this study, was seen in orientation and three-staged command at 12.5%. similar to the study conducted by Kumar et al., showing only 1.8% population showing a reduction in orientation and 0% population showing a reduction in three-staged command [12]. The above data are inferred from the data represented in Figures 1-2.

PAOFI provides information regarding the difficulty the patient experiences in day-to-day activities because of underlying cognitive deficit. The mean score of PLHIV with HAND was recorded at 75.70 ± 19.65, while those without HAND were 190.37 ± 9.89. The significant reduction of score attributes to the difficulty faced by patients in performing day-to-day activities. The above data are inferred from the data represented in Table 7.

Limitations

Further investigation and research in the cognitive domains normative data of Indian population with its vast discrepancies in educational and socioeconomic status. This is recommended amongst the high-risk population of lower socioeconomic class which form a substantial part of PLHIV in India. The clinic diagnosis of HAND if substantiated with biochemical and radiological investigations increase the probability of diagnosis, especially in patients with significant co-morbidities.

## Conclusions

The study conducted reveals a higher prevalence of HIV-associated neurocognitive deficit in patients who were unmarried, received education for less than 12^th^ standard, had a CD4 count <200 cell/mm^3^, and were on cART for less than three years. Use of 3MS indicates maximum involvement in similarities and read and obey amongst all the other tests. PAOFI reveals significant deterioration of the ability to perform daily activities in patients of HAND. These tests, if used in screening HIV-positive patients, can be indicative of underlying cognitive impairment.

## Figures and Tables

**Figure 3:**
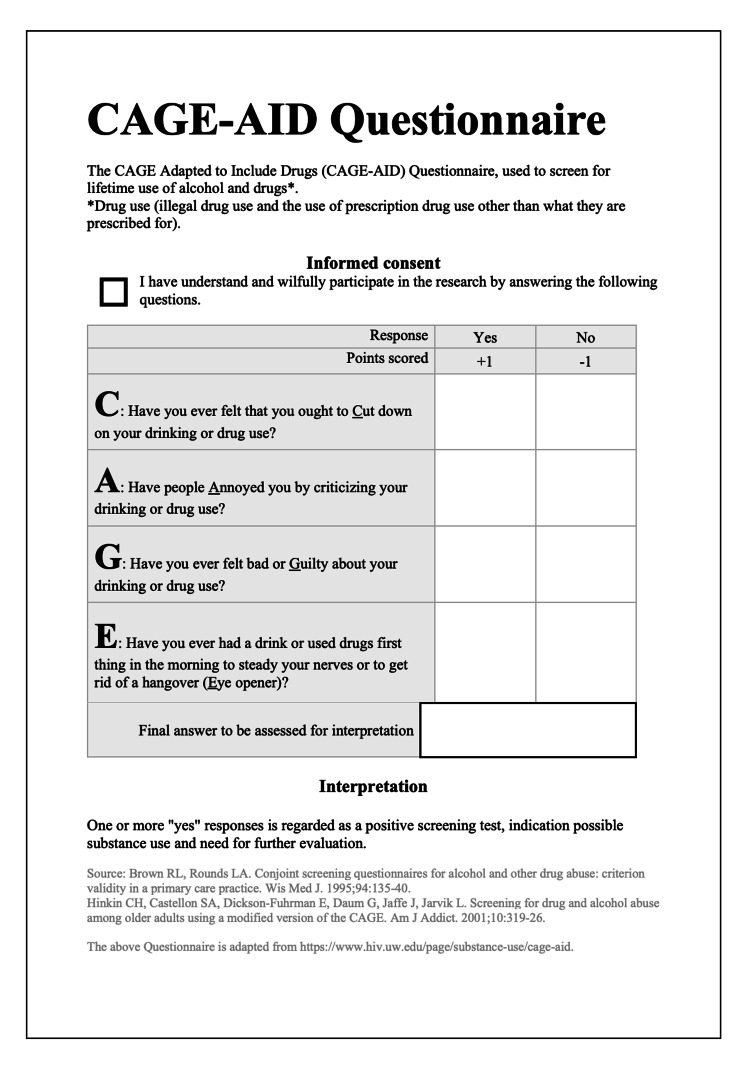
CAGE-AID questionnaire

**Figure 4:**
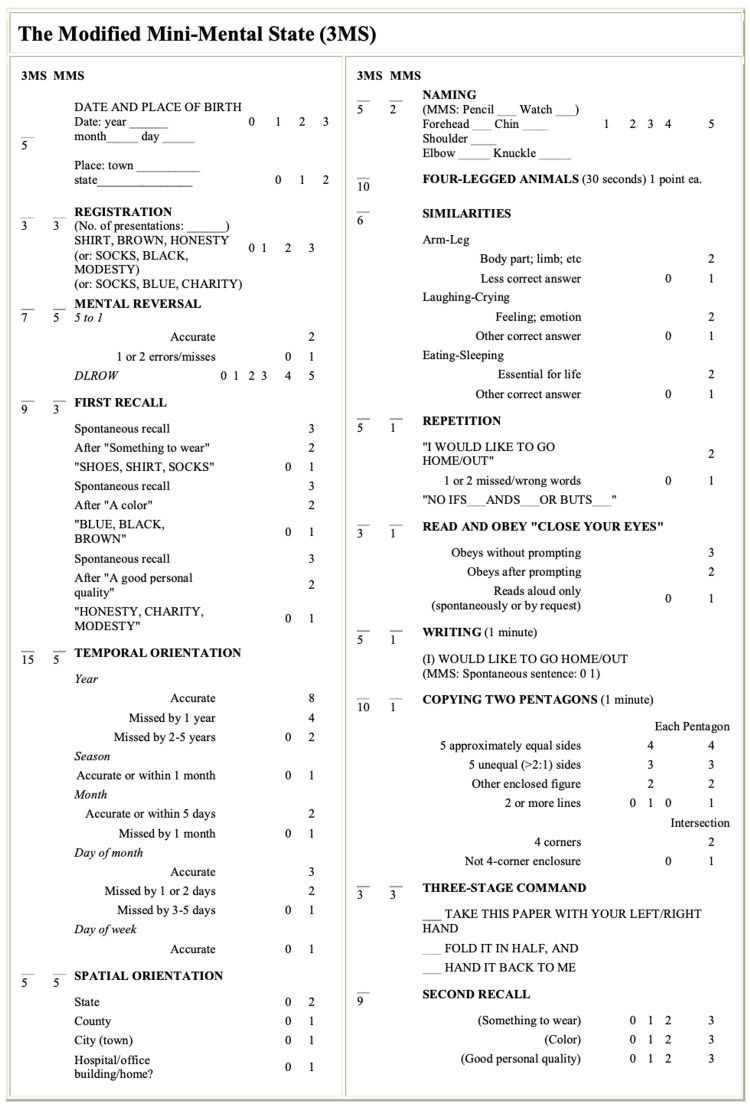
Modified Mini-Mental State Examination form

**Figure 5:**
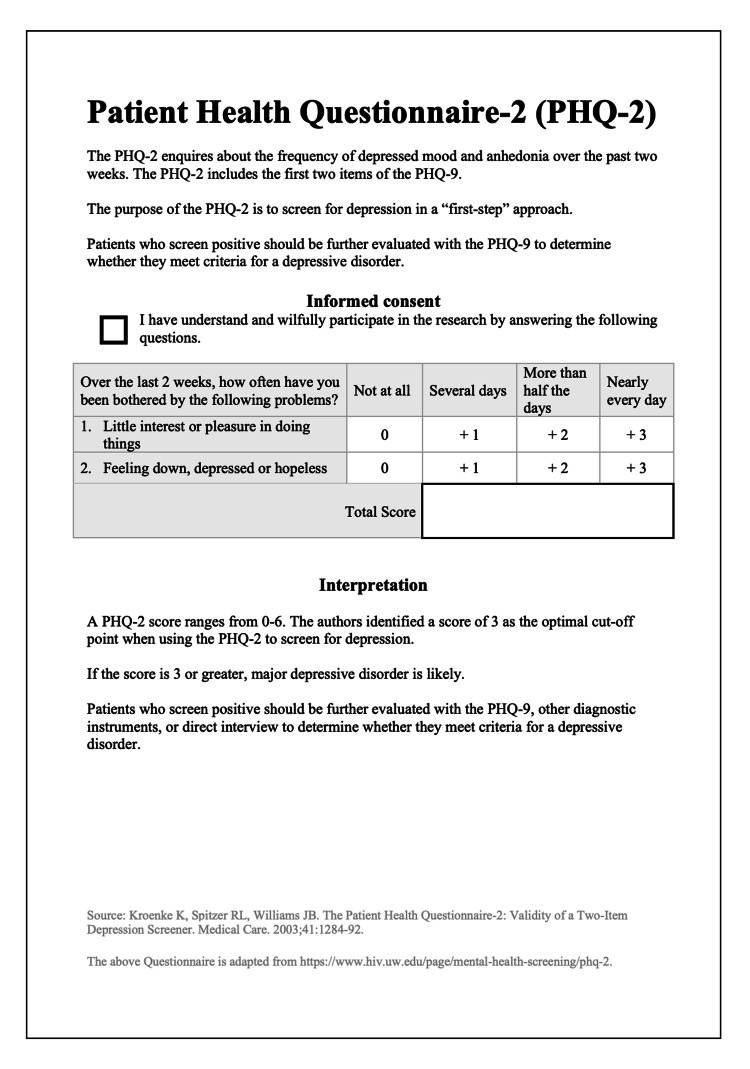
Patient Health Questionnaire-2

**Figure 6:**
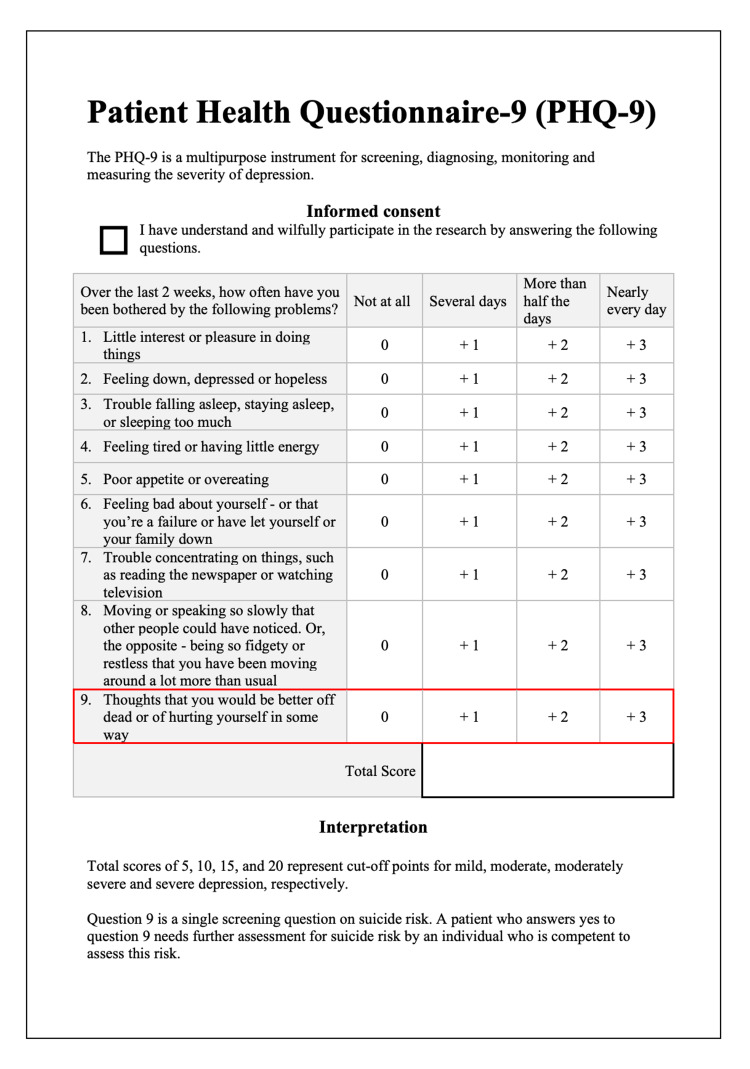
Patient Health Questionnaire-9
